# Antiproliferative and Antitumour Effect of Nongenotoxic Silver Nanoparticles on Melanoma Models

**DOI:** 10.1155/2019/4528241

**Published:** 2019-07-25

**Authors:** Lucía M. Valenzuela-Salas, Nayeli G. Girón-Vázquez, Juan C. García-Ramos, Olivia Torres-Bugarín, Claudia Gómez, Alexey Pestryakov, Luis J. Villarreal-Gómez, Yanis Toledano-Magaña, Nina Bogdanchikova

**Affiliations:** ^1^Escuela de Ciencias de la Salud, Universidad Autónoma de Baja California, Tijuana, Baja California, Mexico; ^2^Facultad de Ingeniería, Arquitectura y Diseño, Universidad Autónoma de Baja California, Ensenada, Baja California, Mexico; ^3^Departamento de Fisicoquímica de Nanomateriales, CONACyT-UNAM-CNyN, Ensenada, Baja California, Mexico; ^4^Programa Internacional de Medicina, Universidad Autónoma de Guadalajara, Zapopan, Jalisco, Mexico; ^5^Department of Technology of Organic Substances and Polymer Materials, Tomsk Polytechnic University, Tomsk, Russia; ^6^Escuela de Ciencias de la Ingeniería y Tecnología, Universidad Autónoma de Baja California, Tijuana, Baja California, Mexico; ^7^Departamento de Fisicoquímica de Nanomateriales, Centro de Nanociencias y Nanotecnología, Universidad Nacional Autónoma de México, Ensenada, Baja California, Mexico

## Abstract

During the last 3 decades, there has been a slow advance to obtain new treatments for malignant melanoma that improve patient survival. In this work, we present a systematic study focused on the antiproliferative and antitumour effect of AgNPs. These nanoparticles are fully characterized, are coated with polyvinylpyrrolidone (PVP), and have an average size of 35 ± 15 nm and a metallic silver content of 1.2% wt. Main changes on cell viability, induction of apoptosis and necrosis, and ROS generation were found on B16-F10 cells after six hours of exposure to AgNPs (IC_50_ = 4.2 *μ*g/mL) or Cisplatin (IC_50_ = 2.0 *μ*g/mL). Despite the similar response for both AgNPs and Cisplatin on antiproliferative potency (cellular viability of 53.95 ± 1.88 and 53.62 ± 1.04) and ROS production (20.27 ± 1.09% and 19.50 ± 0.35%), significantly different cell death pathways were triggered. While AgNPs induce only apoptosis (45.98 ± 1.88%), Cisplatin induces apoptosis and necrosis at the same rate (22.31 ± 1.72% and 24.07 ± 1.10%, respectively). In addition to their antiproliferative activity, *in vivo* experiments showed that treatments of 3, 6, and 12 mg/kg of AgNPs elicit a survival rate almost 4 times higher (*P* < 0.05) compared with the survival rate obtained with Cisplatin (2 mg/kg). Furthermore, the survivor mice treated with AgNPs do not show genotoxic damage determined by micronuclei frequency quantification on peripheral blood cells. These results exhibit the remarkable antitumour activity of a nongenotoxic AgNP formulation and constitute the first advance toward the application of these AgNPs for melanoma treatment, which could considerably reduce adverse effects provoked by currently applied chemotherapeutics.

## 1. Introduction

Melanoma is the most aggressive form of skin cancer and one of the deadliest. The morbidity and mortality of melanoma have increased in recent decades around the world with more than 100,000 new cases reported every year, and the incidence increase continues [1]. The incidence in fair-skinned populations always has the highest growth rates [2], but recently a 500% increase in new cases in Mexico has been reported [3].

In the last 3 decades, there have not been new treatments that efficiently improve patient survival [4]. Melanoma treatment faces two important problems: the adverse effects of chemotherapy due to the lack of selectivity and the low efficiency of the used methods [5]. Even with the resistance and severe adverse effects observed, Cisplatin (CisPt) continues to be the most used drug [6].

In the last decade, inorganic nanoparticles have attracted the interest of several research groups due to their applications in cancer therapy, either as drug nanocarriers or as therapeutic agents [7, 8]. Particularly for the second purpose, silver nanoparticles (AgNPs) have been widely studied. However, their physicochemical properties play a key role in both facets, to fight cancer and to cause adverse effects [9].

In this sense, the employment of polyvinylpyrrolidone (PVP) as a coating agent of AgNPs produces an important decrease in cytotoxic and genotoxic effects compared with noncoated or citrate-AgNPs [10–12]. The PVP-AgNPs could also provide several advantages through selectivity in their antiproliferative action. The commercial formulation of PVP-AgNPs from Skyspring Nanomaterials showed a differential antiproliferative activity between breast triple-negative tumour cells and breast nontumourigenic cells. Besides, the same PVP-AgNP formulation significantly reduces the tumour volume of these aggressive tumours that are difficult to treat [13].

Our research group has studied a commercially available PVP-AgNP formulation known as Argovit™ that has been very effective as an antimicrobial [14, 15], antifungal [15], and antiviral agent [16–18]. This formulation also has been a determining factor in the rapid healing of diabetic foot ulcers [19]. Moreover, these nanoparticles inhibit the growth of human tumour cell cultures of the breast, lung, prostate, cervix, and colon. The main death pathway activated was apoptosis, probably triggered by the overproduction of reactive oxygen species (ROS). There was no necrosis, and neither was there evidence of DNA-damage at the effective inhibitory concentrations determined for each tumour cell line [20].

Considering the growth inhibition activity of these PVP-AgNPs on human tumour cell lines, in this work, we show a systematic study of the *in vitro* and *in vivo* effect of well-characterized AgNPs on murine melanoma models. Cytotoxicity, cell death pathway induction, and ROS generation were analysed *in vitro*. In addition, a subcutaneous melanoma model in C57BL/6J mice was performed following the protocol recommended by the National Institute of Health (NIH) [21], where tumour volume, survival rate, haematological parameters, and genotoxicity of the treatments were evaluated.

## 2. Materials and Methods

### 2.1. Silver Nanoparticles (AgNPs)

Silver nanoparticles used in this work were donated by Dr. Vasily Burmistrov from the Scientific and Production Center Vector-Vita (Russia). Argovit™ is a formulation of PVP-coated AgNPs highly dispersed in water with an overall concentration of 200 mg/mL (20%). The content of metallic silver is 12 mg/mL stabilized with 188 mg/mL of PVP. Dilutions of AgNPs were prepared with distilled and sterile water and were kept at 4°C in darkness.

### 2.2. Silver Nanoparticle Characterization

Dynamic light scattering (DLS) (Malvern Instruments Zetasizer Nano NS model DTS1060; *λ* = 532 nm) was used to determine the hydrodynamic diameter and the zeta potential. Characterization of optical properties was done with the Cary 60 UV-vis spectrophotometer (Agilent Technologies) in the range of 200 to 900 nm. Morphology and size distribution were determined by HR-TEM using a JEOL JEM-2010 microscope. Also, lyophilized AgNPs were characterized by FTIR-ATR in a range of 400 to 4000 cm^−1^ with a resolution of 2 cm^−1^ on a universal diamond ATR Top Plate accessory (PerkinElmer). The sample spectrum was compared with that of standard solid PVP (MW 100 kDa). The silver content of Argovit® was determined by ICP-OES (Varian Vista-MPX CCD Simultaneous ICP-OES).

### 2.3. Cell Culture

B16-F10, murine skin melanoma cells from C57BL/6J mice, were purchased from ATCC® (ATCC® CRL-6475™) and maintained in DMEM high-glucose media (Sigma-Aldrich, 51435C) supplemented with 10% (*v*/*v*) of heat-inactivated Foetal Bovine Serum (FBS, Biowest, S1650) and 2 mM of L-glutamine (Sigma-Aldrich, G5792) at 37°C in a humidified atmosphere with 5% CO_2_. Subculturing was performed every 2 days.

### 2.4. Antiproliferative Activity

B16-F10 cells (1 × 10^5^) were seeded in 96-well plates with 195 *μ*L of DMEM high-glucose supplemented media (Sigma-Aldrich, 51435C). Primary screening was done to determine IC_50_ values using the MTT assay kit (Bio-Vision MTT Cell Proliferation Assay Kit #K299-1000) and ProBit analysis after 24 h of exposure. After that, the IC_50_ of AgNPs (4.2 *μ*g/mL) and Cisplatin (2 *μ*g/mL, CisPt) were employed to determine the cell growth behaviour at 6, 12, 18, and 24 h. The proliferation kinetics was determined by flow cytometry in Attune NxT equipment. Cell viability was determined using the Vybrant™ CFDA SE Cell Tracer Kit and propidium iodide (PI) (Thermo Fisher Scientific, V12883) following the provider's protocol.

Determination of apoptosis and necrosis cell death pathway was determined with the Alexa Fluor® 488 Annexin V/Dead Cell Apoptosis Kit (Thermo Fisher Scientific, V13241). ROS generation was analysed by the DCFDA Cellular ROS Detection Assay Kit (Abcam, 139476) and with the MitoSOX™ Red Mitochondrial Superoxide Indicator for live-cell imaging (Invitrogen, M36008).

### 2.5. Experimental Animals

We purchased seventy 8–10-week-old C57BL/6JNHsd male mice from Envigo, Mexico. Mice were divided and assigned randomly to polycarbonate cages into 6 groups of 10 mice each and two more groups of 5 mice each. The latter groups were used as untreated controls for haematological and genotoxicity trials. Mice were maintained at 25°C temperature, 60% humidity, and 12/12-hour light-dark cycle and fed *at libitum*. The experimental protocol was approved on June 19, 2018, by the Ethical Committee of the Health Sciences School from the Autonomous University of Baja California, Mexico with file number 001/2018.

Melanoma induction in C57BL/6JNHsd mice was performed as recommended by the USA NIH [21]. First, B16-F10 cells in the logarithmic growth phase were harvested for injection; after guaranteeing viability with trypan blue (>90%), cell concentration was adjusted to 1 × 10^6^ cell/mL in ice-cold Hank's Balanced Salt Solution (HBSS, Biowest, L0606).

Mice of 20 g weight were inoculated by subcutaneous administration of 100 *μ*L cell suspension (1 × 10^5^ cells/mouse). The appearance of a “bleb” indicates the correct subcutaneous administration. After cell administration, mice were placed in the corresponding cage. Tumours became palpable in 12 days, and at this point the antitumour trials were initiated.

### 2.6. Antitumour Activity

The mice with palpable tumours (10 per group) were administered subcutaneously each 3rd day for 21 days with the corresponding dose of each treatment as follows: Group 1—negative control (injectable water); Group 2—positive control (CisPt, 2 mg/kg); Group 3—vehicle control (PVP, 12 mg/kg); Group 4—AgNPs, 3 mg/kg; Group 5—AgNPs, 6 mg/kg, and Group 6—AgNPs, 12 mg/kg. Group 7 and Group 8 are healthy mice injected with water or PVP, respectively (*n* = 5 for each group), and used as negative control for the haematological parameters and genotoxicity.

All the AgNP doses were calculated according to the metallic silver content. Dilutions were prepared with injectable water from a stock solution of Argovit® with 12 mg/mL of metallic silver to obtain the work solutions at the final concentrations of 24, 12, and 6 *μ*g/mL. Then, 100 *μ*L was administered to each mouse according to the administration scheme previously described. Doses were chosen due to previous experiences with murine models [17]. The mice were examined daily, and tumours were measured every day of administration with a slide calliper. Tumour volume was determined with the Attia-Weiss formula. Furthermore, general characteristics (activity, hair appearance, and any apparent change) were also recorded.

After 21 days of the administration, the treatment was stopped and the animals observed for 7 more days. After that, the mice were anaesthetized with ketamine and euthanized by cardiac puncture to collect blood samples.

### 2.7. Genotoxicity Determinations

Drop blood samples were taken from the animals before they died (experimental Groups 1, 2, 3, and 5) or euthanized (Groups 4, 6, and 7). Two smears were made on cleaned microscope slides. The smears were air-dried, fixed in absolute ethanol for 10 minutes, and stained with acridine orange (CAS No. 10127023, Sigma-Aldrich).

The micronuclei in each sample were scored manually using a binocular microscope (Carl Zeiss, Axiostar Plus) with a fluorescent filter (IVFL, 450–490 nm). The number of micronucleated erythrocytes (MNE) in 10,000 total erythrocytes and the polychromatic erythrocytes (PCE) in 1000 total erythrocytes were evaluated.

### 2.8. Statistical Analysis

Viability, apoptosis, necrosis, ROS generation, and tumour volume data, were analysed with a two-way ANOVA (*P* < 0.05) followed by the Tukey post hoc test (*P* < 0.05) (GraphPad Prism). For relative survival % data, the Mantel-Haenszel chi-square statistic was used (*P* < 0.05) (GraphPad Prism).

## 3. Results

### 3.1. Silver Nanoparticle Characterization

A complete characterization of the sample provided by Prof. Burmistrov was performed. AgNPs are mainly spherical with an average size of 35 ± 15 nm as determined by HR-TEM. The hydrodynamic diameter (summarized diameter of a metallic silver nanoparticle and PVP coating) determined by DLS was 70 nm, and the *ζ* potential was -15 mV. UV-vis analysis showed an absorption peak at 420 nm that corresponds to the plasmon surface resonance. The silver content of the concentrate solution was 12 mg/mL, determined by ICP-OES.

The FTIR-ATR spectrum shows peaks corresponding to the hydroxyl vibration of unbound water (*ν*OH) at 3406 cm^−1^, carbonyl stretching at 1650 cm^−1^ (*ν*C=O), symmetrical stretching of nitrogen in the ring at 1269 cm^−1^ (*ν*_*s*_C-H), and asymmetric (*ν*_*as*_C-H) stretching at 2948 and 2915 cm^−1^, respectively. Metallic silver determined by ICP-OES is 12 mg/ml (% wt).

### 3.2. Cell Viability, Apoptosis, Necrosis, and Reactive Oxygen Species Quantification *In Vitro*

**Figure 1:**
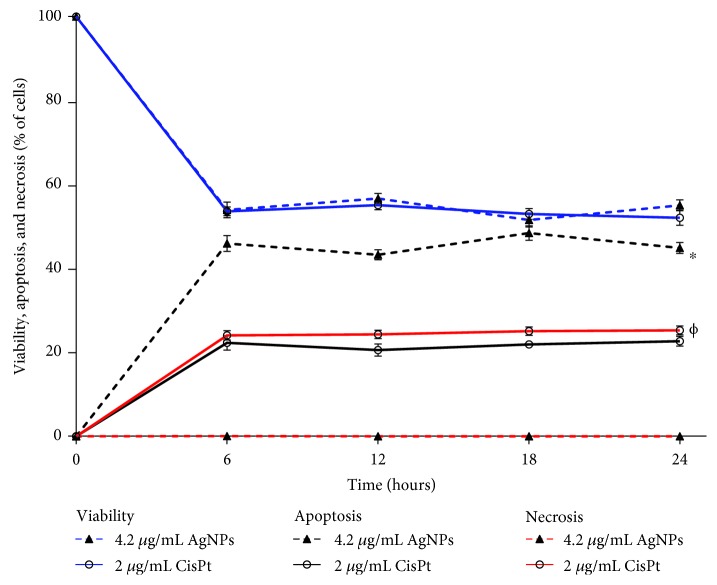
Viability, apoptosis, and necrosis determined in B16-F10 cell cultures treated with AgNPs or CisPt. Determinations were done at 6, 12, 18, and 24 h in melanoma cell cultures treated with the concentrations of AgNPs or CisPt indicated in the graph. Viability, blue lines; apoptosis, black lines; necrosis, red lines. ∗ indicates a statistically significant difference of *P* < 0.05 for apoptosis data. *Φ* indicates a statistically significant difference of *P* < 0.05 for necrosis data.

In this work, cellular viability, induction of apoptosis or necrosis, and ROS generation changes on murine melanoma cells B16-F10 after exposure to IC_50_ of AgNPs (4.2 *μ*g/mL) or CisPt (2.0 *μ*g/mL) were evaluated at 6, 12, 18, and 24 h. The main changes in cellular viability were observed in the first 6 h following the administration of the agents, CisPt or AgNPs. After that, the effect was maintained until the last record at 24 h (Figure 1).

From the record of cellular viability, both agents AgNPs and CisPt present the same antiproliferative effect (53.95 ± 1.88 and 53.62 ± 1.04, respectively), but the cell death pathways induced are quite different. After 6 h of exposure, 45.97 ± 1.88% of apoptosis was observed on cells treated with AgNPs, twofold higher than the apoptotic cells found when exposed to CisPt (22.31 ± 1.72%, Figure 1). On the other hand, treatment with AgNPs produces less than 0.1% of necrosis, while for CisPt it was 24.06 ± 1.09%. For CisPt, both cell death pathways were induced practically at the same rate (Figure 1). Behaviour found after 6 h of exposure was maintained until the final determination was made at 24 h of exposure. Complete data of cellular viability, apoptosis, and necrosis can be consulted in the supplementary material Table S1.

**Figure 2:**
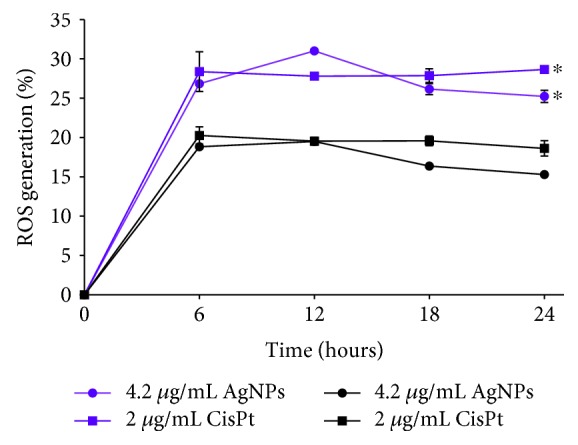
Reactive oxygen species production in melanoma cells treated with IC_50_ of AgNPs or CisPt. Quantification of ROS production in B16-F10 cell cultures treated with the IC_50_ of AgNPs or CisPt was performed by flow cytometry and fluorescent markers. Total ROS was determined with DCFDA (purple lines) and mitochondrial superoxide with MitoSOX (black lines). ∗ indicates a statistically significant difference of *P* < 0.05 comparing total ROS generation data with mitochondrial superoxide data.

The most significant reactive oxygen species (ROS) generation was found after exposure of 6 h to CisPt or AgNPs (Figure 2) and shows an inverse relationship with cellular viability. Cells treated with CisPt reached the maximal ROS production after 6 h of exposure (20.27 ± 1.09%); meanwhile, the highest ROS production induced by AgNPs was observed at 12 h (19.50 ± 0.35%).

The mitochondrial superoxide production has the same behaviour through time with that described for total ROS and corresponds to two-thirds of the total amount of ROS observed with 31.02 ± 0.45% for AgNPs and 28.38 ± 2.52% for CisPt. The most important difference between the treatments is that CisPt induces a sustained ROS generation after 6 h and until 24 h, while a decrease in ROS generation was observed after 12 h of exposure when melanoma cells were treated with AgNPs (Figure 2). All data of ROS production through time can be consulted in supplementary material Table S2.

### 3.3. Antitumour Activity

Tumour volume, as well as signs of illness, were observed throughout the experiment after the treatment administration described in Materials and Methods. Tumour volume increased in all mice with or without treatment. Mice with tumours treated with AgNPs did not stop eating or drinking through the whole experiment. Mice with the tumour but untreated, as well as those treated with CisPt or PVP, presented lethargy and loss of appetite.

**Figure 3:**
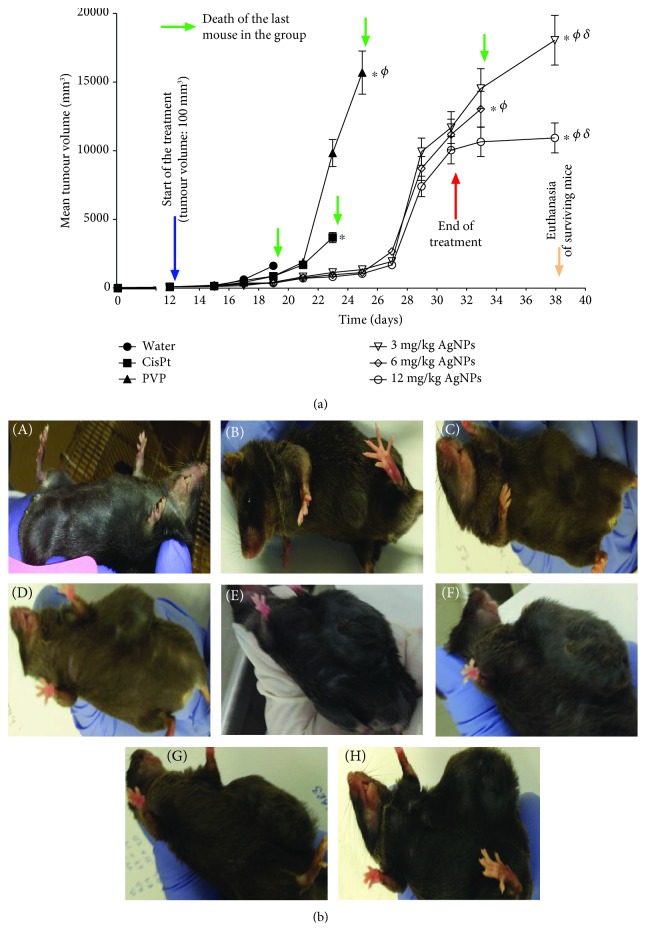
(a) Mean melanoma tumour volume as a function of time (days). Mice treated with injectable water were used as negative controls (water); mice treated with 2 mg/kg of Cisplatin (CisPt), 12 mg/kg of PVP (PVP), and 3, 6, or 12 mg/kg of AgNPs are indicated in the figure. Melanoma cells were inoculated subcutaneously at the beginning of the experiment (black arrow); treatments start when tumours were 100 mm^3^ (blue arrow). Treatments ended after 21 days (red arrow). Tumour volume determination was stopped when all mice in the group died (green arrows). 7 days after the end of the treatments (yellow arrow), surviving mice were euthanized. ∗ indicates a statistically significant difference of *P* < 0.05 comparing groups with the group treated with water. *Φ* indicates a statistically significant difference of *P* < 0.05comparing groups with the group treated with CisPt. *δ* indicates a statistically significant difference of *P* < 0.05 comparing between AgNP doses. (b) Pictures showing mouse without melanoma (A), when tumour reached 100 mm^3^, and when this tumour volume was reached in mice with the following corresponding treatments (C-H): (C) water, (D) CisPt, (E) PVP, and (G) 6 mg/kg. The surviving mice were euthanized on the 38th day: (F) AgNP 3 mg/kg and (H) AgNP 12 mg/kg. The last mice that died are marked with green arrows in (a).

Mice injected with water developed tumours of around 1500 mm^3^ and died 7 days after the tumour become palpable. The tumour volume in animals treated with PVP or CisPt presented a similar tumour volume (around 1500 mm^3^) after 9 days of the start of treatment (21st day in Figure 3). On the other hand, mice treated with AgNP doses of 3, 6, and 12 mg/kg exhibited a tumour volume within the range of 722-837 mm^3^. This represents a 50-60% tumour growth inhibition elicited by the AgNP treatments compared with PVP (1,890 mm^3^), CisPt (1,704 mm^3^), or water (1,500 mm^3^). It is important to recall that all mice injected with water died on the 7th day of treatment (Figure 3).

For the 11th day of treatment (day 23 in Figure 3), while the tumour growth rate in animals treated with any of the three doses of AgNPs increases in a quite similar way (tumour volume in the range of 837-1,142 mm^3^), animals treated with CisPt or PVP showed tumours with a volume of 3,500 and 9,500 mm^3^, respectively. These values represent a 72% reduction in tumour growth caused by the administration of AgNPs compared with CisPt and a 90% reduction compared with PVP.

The evaluated doses of AgNPs did not show differences between them regarding tumour growth until the 19th day of treatment, which also was the last day of treatment. However, in the last seven days of the experiment (the 32nd-38th day in Figure 3), sharp differences were observed. The last surviving mouse of the group treated with 6 mg/kg died 2 days after the end of the treatment (day 33 in Figure 3), while on the group of mice treated with 3 and 12 mg/kg of AgNP, a mouse of each group survived the 7 days of the observation period.

Surviving mice of the experimental groups treated with 3 and 12 mg/kg of AgNPs show tumour volumes at the end of the observational period of 18,000 and 11,000 mm^3^, respectively (on the 38th day of Figure 3). Thus, it seems that a dose of 12 mg/kg of AgNPs inhibited the tumour growth by almost 50% compared with the treatment of 3 mg/kg (Figure 3), which strongly suggest a dose-dependent behaviour.

**Figure 4:**
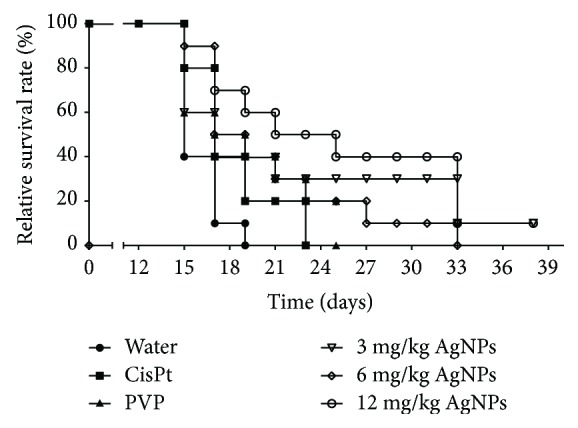
Kaplan-Meier plot showing the relative survival rates of mice treated with AgNPs or CisPt. Mice without melanoma and without treatment were used as negative controls (healthy mice, 100% survival rate). Mice with melanoma and injected with water (water), 2 mg/kg of Cisplatin (CisPt), 12 mg/kg of PVP (PVP), and 3, 6, or 12 mg/kg of AgNPs are identified at the bottom of the figure. ∗ and *Φ* indicate a statistically significant difference of *P* < 0.05 comparing groups treated with AgNPs with groups treated with water and CisPt, respectively.

Figure 4 schematically shows the relative survival rate of mice treated with the different agents. On the 11th day of treatment (day 23 in Figure 4), the death of the last mouse of the group treated with CisPt was registered, while 50% of the mice treated with 12 mg/kg of AgNPs were still alive. At the end of the treatment scheme (day 33 in Figure 4), the life expectancy of the surviving individuals treated with AgNPs increased by 12, 8, and 6 days compared with those injected with water, CisPt, or PVP, respectively (Figure 4). At the end of the experiment, mice treated with 3 or 12 mg/kg of AgNPs survived 19, 15, and 13 days more than those treated with water, CisPt, or PVP, respectively. This represents a life expectancy of mice treated with AgNPs almost 4-times higher compared with those injected with water, and more than double compared with those mice treated with 2 mg/kg of CisPt (Figure 4). In our knowledge, this is the first time that those effects are observed with an AgNP treatment on an *in vivo* melanoma model.

### 3.4. Haematological Parameters

To complete the profile of the survivor animals to the treatment scheme (mice of groups treated with 3 and 12 mg/kg), their haematological parameters were compared with those observed in healthy mice injected with water or PVP (negative control groups, *n* = 5 for each group).

**Figure 5:**
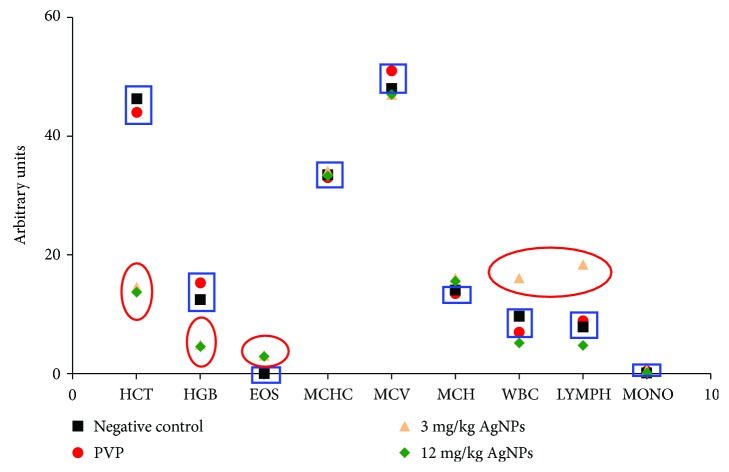
Haematological parameters in the surviving animals at the end of the study. Haematocrit (HCT), haemoglobin (HGB), eosinophils (EOS), mean corpuscular volume (MCV), mean corpuscular haemoglobin concentration (MCHC), white blood cells (WBC), lymphocytes (LYMPH), and monocytes (MONO) were determined in surviving mice treated with AgNPs and in mice without melanoma injected with water or PVP. HCT, HGB, WBC, and LYMPH were out of the range reported as normal (blue boxes) and those outside the normal ranges in mice treated with AgNPs (red circles).

In general terms, the parameters of individuals treated with AgNPs were within the ranges considered as normal for these mice [22], with the exception of haematocrit (HCT) and haemoglobin (HGT), where an important decrease was observed for both groups treated with 3 and 12 mg/kg of AgNPs. Besides, an increase on white blood cells and lymphocytes was observed, but only in the mouse treated with the AgNP dose of 3 mg/kg (Figure 5).

### 3.5. Genotoxicity

Finally, the quantification of micronuclei on erythrocytes (MNE) in 10,000 of the total erythrocytes that indicate genotoxic damage and the counting of polychromatic erythrocytes (PCE) in 1000 total erythrocytes that indicate myelosuppression (cytotoxic effect) were performed.

Tail blood samples from healthy mice, surviving mice of the AgNP treatments of 3 and 12 mg/kg, and from the last survivor of the other treatment groups (untreated controls with melanoma and mice with melanoma injected with PVP, CisPt, and AgNP 6 mg/kg, respectively) were used to identify genotoxic and cytotoxic effects of the treatments.

**Figure 6:**
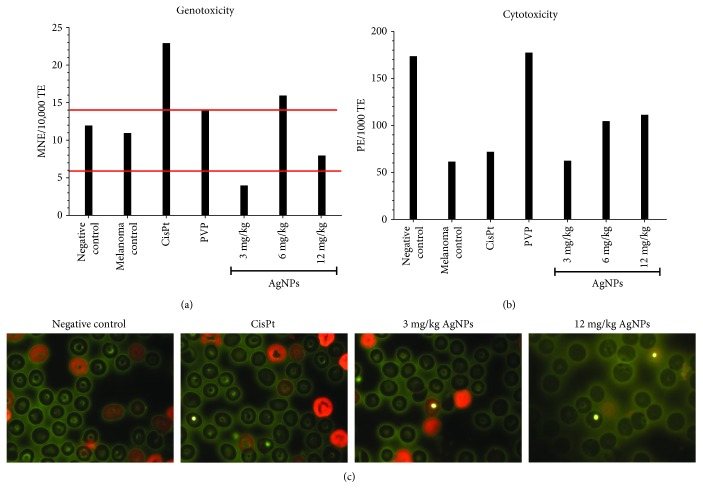
Cytotoxic and genotoxic effects quantified on erythrocytes from circulating blood samples. (a) Average of micronucleated erythrocytes (MNE) scored in 10,000 of total erythrocytes, (b) polychromatic erythrocytes (PCE) in 1000 erythrocytes, and (c) representative images of scored polychromatic erythrocytes (polychromatic erythrocytes, red; normochromatic erythrocytes, green; micronuclei, yellow). Healthy mice (negative control), untreated mice (melanoma control), and mice with melanoma treated with CisPt, PVP, and the indicated doses of AgNPs. Red lines indicate the normal micronuclei range reported for C57BL/6J mice, polychromatic erythrocytes (red), normochromatic erythrocytes (green), micronuclei (yellow).

Results show that mice with melanoma present practically the same MNE count as that of healthy mice, both within the range of 6-14 MNE reported for this strain [23] (marked with red lines in Figure 6). CisPt produces a higher number of MNE with an MNE count of 23. Interestingly, the dose of 6 mg/kg of AgNP generates a higher count of MNE than the upper limit reported. PVP produces 14 MNE, while AgNP doses of 3 and 12 mg/kg produce 4 and 8 MNE, respectively.

On the other hand, it is clear from Figure 6 that the counting of PCE in the mouse with melanoma is substantially lower than that found in the healthy one. For CisPt, a small increase compared with the positive control was found, while a dose-dependent behaviour was observed for AgNP doses; however, none of them are comparable with the effect observed for PVP, which practically overturned the myelosuppression promoted by melanoma and reached PCE levels like those found in the healthy mice.

## 4. Discussion

During the past decades, metal nanoparticles [24–26] and particularly AgNPs ([27]) have shown a real potential to inhibit tumour cell proliferation. It has been published that AgNP toxicity could be related with the release of Ag^+^ ions [28–30] or to the whole nanoparticles [28, 29] which induce ROS generation, modifying the transmembrane potential of mitochondria and, in turn, trigger the activation of several cell death pathways.

The physicochemical properties of AgNPs such as size, coating, and metal content have been related with their cytotoxic effect in mammalian cells [31–33]. In human cell lines, the size of AgNPs plays a key role in the effects on viability, membrane integrity, and ROS generation [32, 34–36]. Specifically, a smaller size of AgNPs induce a higher cytotoxicity [37–40]. On the other hand, coating agents provide different stability degrees that directly influence the cytotoxic and genotoxic effects [11, 41, 42].

The effect of the AgNP formulation studied on this work on the B16-F10 murine melanoma cells with an IC_50_ of 4.2 *μ*g/mL after 24 h of exposure is quite similar to that found for human tumour cell lines of the cervix (HeLa), breast (MDA-MB-231 and MCF-7), prostate (DU-145), colon (DLD-1 and HT-29), and lung (H-1299 and H-1437). For all of them, the IC_50_ values found were within the concentration range of 1.82 to 3.43 *μ*g/mL after the same exposure time. As found for B16-F10 in this work, in all types of the tumour cells evaluated, the main cell death pathway induced is apoptosis and the cellular viability showed an inverse relationship with ROS overproduction. At the IC_50_ value determined for each tumour cell, no DNA damage was found according to the comet assays performed [20].

Interestingly, according to the proliferation kinetics, the main changes elicited by AgNPs on B16-F10 cells were not produced at 24 h but only after 6 h of exposure (Figure 1). After this time of exposure, cell viability and ROS overproduction have shown an inverse relationship (Figure 2). Besides, apoptosis reached its highest levels without necrosis evidence (Figure 1). Consequently, AgNPs need only 6 h to provoke the cellular damage that leads melanoma cells to die by apoptosis (Figures 1 and 2).

Despite that both the agents AgNPs and CisPt produce similar effects on melanoma cells regarding cell viability and ROS overproduction, the final consequence is quite different. While the former induces only apoptosis as the main cell death pathway, the latter induces practically the same amount of apoptosis and necrosis.

On *in vitro* conditions, the CisPt-DNA adduct can be formed after 1-3 h in the blood cells and tumour tissue of cancer patients [43]. Thus, the quick obtainment of the CisPt-DNA adduct, the decrease of glutathione (GSH) [44], and the production of ROS in the mitochondria that, in turn, collapses energy production [45] could be independent of concomitant factors that contribute to the presence of 40 times more necrosis after CisPt administration compared with exposure to AgNPs.

Although the molecular mechanism of cytotoxicity elicited by this AgNP formulation is still not fully elucidated, these results represent an important advantage for AgNPs compared with CisPt because necrotic cellular debris promotes a proinflammatory response that is associated with tissue damage, processes not observed with the induction of programmed cell death ([46]).

Furthermore, the antitumour activity observed in mice treated with CisPt is completely different from that observed with AgNP treatments. The animals treated with CisPt died on the 11th day after the start of the treatment. During this time, they show lethargy and loss of appetite. This symptomatology could be related to the rapid and uncontrollable damage produced at the cellular level, which could be promoted by ROS overproduction in mitochondria and the alteration of the DNA structure that, finally, triggers necrosis as the main cell death pathway.

Unlike the treatment with CisPt, the AgNP treatments achieved a remarkable survival time with the three concentrations assayed. At least one mouse of the groups treated with 3, 6, or 12 mg/kg of AgNPs reached the 21st day of the treatment alive; this represents almost a quadruplication of the lifespan compared with that of mice injected with water and doubling the lifespan compared with that of mice treated with CisPt.

As far as we know, there are only two papers that analyse the effect of AgNPs in an *in vivo* melanoma murine model. In the first work [47], a study of 17 days was performed using wortmannin, AgNPs, or a combination of both. Daily administration for 5 days starting with a tumour volume range of 50-100 mm^3^ was used. Animals were euthanized eight days after the end of the treatment (13 days of observation).

This treatment scheme is quite different from that recommended for the NIH, but it is important to note that the authors of the work were focused in the modulation of autophagy to enhance the antiproliferative effect of AgNPs against cancer cells [47]. In general, Lin et al. found a much lower tumour growth inhibition than what we found in the same time window (thirteen days after starting treatment).

The largest tumour volume they found in their controls on day 13 after the treatment began is about 1200 mm^3^; those treated with their AgNPs had a tumour volume close to 500 mm^3^ and those treated with the combination of AgNPs and wortmannin had a tumour volume of around 300 mm^3^. In this work, we found that on the 13th day after treatment began mice injected with water and CisPt already died; meanwhile, the surviving mouse of the group injected with PVP had a tumour volume of 9500 mm^3^ and all mice treated with AgNPs presented tumour volumes between 800 and 1150 mm^3^ (Figure 3). Unfortunately, these are not conclusive data due to the differences on the initial tumour volume, dosage frequency, days of treatment, follow-up days, and an unknown silver content of the AgNPs used by them which do not allow us to make a direct comparison with the results found in this work.

The second article [48] is quite different because it is focused on the angiogenic capacity of AgNPs. The authors report that B16-F10 melanoma cells exposed to AgNPs and then injected intradermally in C57BL/6J mice induced angiogenesis in the area near to the tumour and increased the haemoglobin concentration within the tumour. Smaller nanoparticles (2 nm, Table 1) elicited the vascularization around the tumour in the melanoma model, and the angiogenic effect is enhanced if the cells were previously exposed to AgNPs. The angiogenic effect elicited by low doses of AgNPs could promote the tumour growth, but this is reversed when the dose or the size of the nanoparticle increase [48].

On the other hand, AgNPs with a higher size (53 nm, Table 1) induce autophagy in melanoma cells with high concentrations or induce cell survival with lower concentrations. These works, including ours, are examples of the great impact that the size of nanoparticles with the same stabilizing agent has on murine melanoma. Unfortunately, the same cannot be said for the concentration of silver present in each study due to the lack of information (Table 1). The main similarities and differences between the characterization of AgNPs previously published and the AgNPs used in this work in an *in vivo* melanoma model are shown in Table 1.

A very important result from this work is that at the higher dose of AgNPs tested (12 mg/kg) the tumour growth seems to be inhibited, since the tumour volume remains constant during the whole observational period (seven days); that is, from the 31st to the 38th day of the experiment (Figure 3).

This response must necessarily be related to the dose-dependent behaviour observed on the EPC counting that could be interpreted as a lower myelosuppression effect as the AgNP doses increase. Also, this is consistent with the general behaviour of mice treated with AgNPs that continued to be active and kept on feeding, in spite of the size of the tumour. This is contrary to the behaviour observed on mice treated with CisPt and those from the negative control group (Figure 6). These results suggest a protective dose-dependent effect elicited by AgNP treatments (Figure 6).

From Figure 6, it is clear that the higher protection against the myelosuppressive effect is provided by the PVP. However, only the combination of PVP and metallic silver to obtain the AgNP formulation possesses both the protective effect against myelosuppression and the antitumour activity.

Moreover, no genotoxic effect was observed on AgNP treatments of 3 and 12 mg/kg, both with an MNE counting below the upper limit of the range reported for this mouse [23] (marked in Figure 6 with red lines). Mice from the positive control group (with melanoma and without treatment) showed an MNE count within the marked range as well as the mice treated with PVP. In this sense, given that it has been reported that rodents are more sensitive to the genotoxic damage induced by AgNP than humans [49], one would expect very low damage caused by the administration of the AgNP formulation studied here in humans.

Conversely, samples from the last mouse of the group treated with CisPt showed almost double the MNE. This could be associated with the necrosis induction and the limited survival time observed on mice treated with this compound. It is known that platinum-based drugs, among them CisPt, are genotoxic. This fact is related with the appearance of new tumours and drug resistance, which means that, even when the CisPt treatment is effective against a tumour, there is an oncogenic risk due to its genotoxic effect [50].

Regarding the behaviour observed for mice treated with 6 mg/kg of AgNPs, these experiments do not provide enough arguments to explain the death of the last mouse five days earlier than the other surviving animals treated with 3 and 12 mg/kg of AgNPs, respectively (Figure 6). But, as in the case of CisPt, the higher amount of MNE compared with basal values could be associated with the death of that mouse. Further experiments must be done to clarify this point.

Surviving mice present haematological parameters quite similar to that found in healthy mice [22], but the increases observed in WBC and LYMPH with the lowest dose of AgNPs (3 mg/kg) are consistent with the activation of immunological system cells by the low concentration of AgNPs [51]. On the other hand, the lower levels of haemoglobin (HGB) and haematocrit (HCT) on surviving mice treated with both 3 and 12 mg/kg of AgNPs can be interpreted as anaemia.

It has been reported that nanosized colloidal silver stabilized with PVP may induce HCT and HGB decrease [52], and other AgNPs promoted venous thrombus formation by platelet aggregation [53]. However, in this case, the observed decrease in HCT and HGC could be attributable to the development of melanoma itself. As shown in the Results, mice with melanoma but without treatment showed an important decrease on PCE counting. Therefore, the low levels of HCT and HGB could be the result of cumulative damage due to the progress of the disease and not because of a toxic effect of the administered AgNP.

For the purposes of avoiding as much as possible the anaemia in further studies, a systemic iron supplementation is proposed. It has been reported that this could be more effective than a twofold iron diet [54]. Also, it is known that a deficiency of other elements such as copper or selenium could be involved in the anaemia process [55, 56], but this is out of the scope of this work, and further analysis needs to be done to identify the cause and to prevent the anaemia.

Therefore, this work presents a systematic study for evaluating the antitumour effect of AgNPs in melanoma under standardized conditions, following NIH recommendations, and for providing the complete characterization of AgNPs, specifically the concentrations and doses of the active component, metallic silver. As a consequence, apoptosis induction, antitumour activity, lifespan increase, the absence of genotoxicity in blood samples, and the observed protective effect against myelosuppression elicited by melanoma on mice that have been exposed during 21 days to AgNPs is an irrefutable probe of the highest biocompatibility of these AgNPs compared with CisPt. All these results suggest that the possible adverse effects elicited by these AgNPs in humans might be less than the already known effects promoted by CisPt. This emphasizes the potential of AgNPs as an alternative for cancer treatment and urges the completeness of their preclinical studies.

## 5. Conclusions

This work is a systematic approach to evaluate the antiproliferative and antitumour effect of AgNPs in melanoma under standardized conditions following the recommended protocol by NIH. The AgNP formulation studied in this work, Argovit®™, possesses a higher antitumour activity and biocompatibility on C57BL/6JNHsd mice inoculated with murine melanoma B16-F10 cells than that found in one of the most employed chemotherapeutic agents in melanoma treatment, CisPt.

The higher biocompatibility of these AgNPs compared with CisPt is manifested *in vitro* as apoptosis induction, which is the main cell death pathway after 6 h of exposure triggered by ROS overproduction, mainly on mitochondria. Meanwhile, their high capacity to reduce tumour growth, remarkable lifespan increase (quadruple compared with nontreated and double compared with those treated with CisPt), lack of genotoxic damage, and the possible protective effect against myelosuppression, elicited by the natural progression of melanoma, were observed in *in vivo* assays.

These findings show the importance of adequate physicochemical characteristics, such as size (35 nm), the optimal content of metallic Ag, and the effective metallic Ag/PVP ratio which provides AgNPs a high stability, to elicit the tumour growth rate decrease and to increase the life expectancy in individuals with one of the most aggressive skin cancers known. All these can be gained without evident genotoxic effects and even decreasing the myelosuppression provoked by the natural progression of the disease, urging the completeness of its preclinical studies.

## Figures and Tables

**Table 1:** Physicochemical properties of AgNPs employed on *in vivo* murine melanoma models.

Property	Lin et al. [47]	Kang et al. [48]	This work
AgNP size (nm)	58.8 ± 1.7	2.3	35 ± 15
Protective agent	PVP	PVP	PVP
*Z* potential (mV)	−12.9 ± 1.1	-0.28	-15
Metallic silver content (% wt)	ND^∗^	ND^∗^	1.2
Initial tumour diameter (mm)	4-6	NA^†^	10
Used dose: metallic silver (mg/kg)	1.5	1, 5, and 10	3, 6, and 12
Days of administration in treatment	5	NA^†^	21
Dosage frequency	24 h	NA^†^	48 h
Days without treatment before sacrifice	8	NA^†^	7

^∗^ ND: not determined. ^†^NA: not applicable.

## Data Availability

The data used to support the findings of this study are available from the corresponding author upon request.

## Supplementary Materials

Supplementary Materials Table S1: (related to Figure 1). Cellular viability, apoptosis, and necrosis recorded on B16-F10 cultures through time. Table S2: (related to Figure 2). ROS quantification by DCFDA and MitoSOX on B16-F10 exposed to AgNP and Cisplatin.
