# Supplementary material for: Antiproliferative and Antitumour Effect of Nongenotoxic Silver Nanoparticles on Melanoma Models
Source: Oxid Med Cell Longev. 2019 Jul 25;2019:4528241. doi: 10.1155/2019/4528241 (PMC6683800; doi:10.1155/2019/4528241)
Supplement: Supplementary Materials — Table S1: (related to Figure 1). Cellular viability, apoptosis, and necrosis recorded on B16-F10 cultures through time. Table S2: (related to Figure 2). ROS quantification by DCFDA and MitoSOX on B16-F10 exposed to AgNP and Cisplatin. [file 4528241.f1.docx]

Supplementary information

# Antiproliferative and antitumor effect of non-genotoxic silver nanoparticles on melanoma models

Lucía M. Valenzuela-Salas,^1#^ Nayeli G. Girón-Vázquez,^2#^ Juan C. García-Ramos,^3^ Olivia Torres-Bugarín,^4^ Claudia Gómez,^2^ Alexey Pestryakov,^5^ Luis J. Villarreal-Gómez,^6^ Yanis Toledano-Magaña,^3*^ Nina Bogdanchikova^7^

Table S1. Cellular viability, apoptosis and necrosis recorded on B16-F10 through time

| **Time (hours)** | **Cellular Viability** | | | | **Apoptosis** | | | | **Necrosis** | | | |
| --- | --- | --- | --- | --- | --- | --- | --- | --- | --- | --- | --- | --- |
|  | 4.2µg/ml AgNPs | | 2µg/ml CisPt | | 4.2µg/ml AgNPs | | 2µg/ml CisPt | | 4.2µg/ml AgNPs | | 2µg/ml CisPt | |
|  | Mean | SD | Mean | SD | Mean | SD | Mean | SD | Mean | SD | Mean | SD |
| 0 | 100.00 | 0.00 | 100.00 | 0.00 | 0.00 | 0.00 | 0.00 | 0.00 | 0.00 | 0.00 | 0.00 | 0.00 |
| 6 | 53.96 | 1.88 | 53.62 | 1.05 | 45.98 | 1.88 | 22.31 | 1.72 | 0.07 | 0.00 | 24.07 | 1.10 |
| 12 | 56.69 | 1.23 | 55.11 | 1.10 | 43.29 | 1.23 | 20.58 | 1.43 | 0.02 | 0.00 | 24.31 | 1.01 |
| 18 | 51.54 | 1.72 | 52.99 | 1.32 | 48.45 | 1.72 | 21.93 | 0.39 | 0.01 | 0.00 | 25.08 | 1.01 |
| 24 | 55.04 | 1.30 | 52.09 | 1.77 | 44.92 | 1.31 | 22.66 | 1.16 | 0.04 | 0.00 | 25.25 | 1.08 |

Table S2. ROS quantification by DCFDA and MitoSox

| **Time (hours)** | **ROS in mitochondria** | | | | **Total ROS** | | | |
| --- | --- | --- | --- | --- | --- | --- | --- | --- |
|  | 4.2µg/ml AgNPs | | 2µg/ml CisPt | | 4.2µg/ml AgNPs | | 2µg/ml CisPt | |
|  | Mean | SD | Mean | SD | Mean | SD | Mean | SD |
| 0 | 0.00 | 0.00 | 0.00 | 0.00 | 0.00 | 0.00 | 0.00 | 0.00 |
| 6 | 26.84 | 0.10 | 28.38 | 2.52 | 18.84 | 0.40 | 20.27 | 1.09 |
| 12 | 31.02 | 0.45 | 27.82 | 0.30 | 19.50 | 0.35 | 19.55 | 0.49 |
| 18 | 26.16 | 0.70 | 27.88 | 0.86 | 16.38 | 0.33 | 19.58 | 0.62 |
| 24 | 25.23 | 0.78 | 28.65 | 0.39 | 15.30 | 0.17 | 18.62 | 0.98 |
